# Flow-dependent regulation of genome-wide mRNA and microRNA expression in endothelial cells *in vivo*

**DOI:** 10.1038/sdata.2014.39

**Published:** 2014-10-28

**Authors:** Sandeep Kumar, Chan Woo Kim, Dong Ju Son, Chih Wen Ni, Hanjoong Jo

**Affiliations:** 1 Wallace H. Coulter Department of Biomedical Engineering, Georgia Institute of Technology and Emory University, 1760 Haygood Drive, HSRB E170, Atlanta, Georgia 30322, USA; 2 School of Applied Biosciences, Kyungpook National University, Daegu 702-701, South Korea; 3 Department of Biomedical Engineering, Khalifa University of Science, Technology and Research, Abu Dhabi, UAE; 4 Division of Cardiology, Emory University, Atlanta, Georgia 30322, USA

## Abstract

Atherosclerosis preferentially occurs in arterial regions exposed to disturbed blood flow (*d-flow*), in part, due to alterations in gene expression in the endothelium. While numerous *in vitro* studies have shown how anti-atherogenic flow and pro-atherogenic flow differently regulate gene expression of cultured endothelial cells, similar *in vivo* studies have been scarce. Recently, we developed a mouse model of atherosclerosis that rapidly develops robust atherosclerosis by partially ligating the left carotid artery (LCA) branches, while using the contralateral right carotid (RCA) as control. We also developed a novel method to collect endothelial-enriched RNAs from the carotids of these animals, which enabled us to perform genome-wide expression analyses of mRNAs and miRNAs in the arterial endothelium exposed to either *d-flow* or *s-flow*. These microarray results were used to identify novel mechanosensitive genes such as DNA methyltransferase-1 and miR-712 that play key roles in atherosclerosis. Here, we report these endothelial mRNA and miRNA expression profiles with in-depth information on experimental procedures along with an example of usage of these data.

## Background & Summary

Atherosclerosis preferentially occurs in arterial regions exposed to disturbed blood flow (*d-flow*), in part, due to alterations in gene expression^1–5^. Vascular endothelial cells respond to blood flow through mechanosensors which transduce the mechanical force associated with flow (known as shear stress) into cell signaling events and ultimately, changes in gene expression^6–8^. *D-flow*, which occurs in branched or curved arteries, is characterized by complex flow patterns with low magnitude and oscillatory shear stress (OS), whereas stable flow (*s-flow*) is characterized by high magnitude laminar shear stress (LS) due to blood flow in the straight sections of the vasculature. *D-flow* and *s-flow* act as antagonistic stimuli to either promote or suppress atherogenesis, respectively, through differential regulation of pro-atherogenic and/or atheroprotective genes^2,9,10^.

Comparison of *in vivo*, *ex vivo*, and *in vitro* endothelial gene expression profiles indicates that *in vivo* flow-sensitive genes appear to be lost or dysregulated when the endothelial cells are adapted into in *vitro* culture systems^2,11^. Previous attempts to study the role of *d-flow* in atherosclerosis gave inconsistent results, as most of the studies were carried out using *in vitro* settings^12–15^. Although these *in vitro* studies have provided critical insights into shear-sensitive mechanisms in cultured endothelial cells using modeled flow conditions, it cannot be assumed that *in vitro* flow-sensitive genes and pathways would be involved in the *in vivo* flow-sensitive vascular response and disease. Therefore, it is critical to study how the arterial endothelium responds to different flow conditions *in vivo*. Although, flow-dependent site-specific endothelial phenotype and the change in gene expression was reported in pigs^7,16,17^. However, an experimental model where a robust and reproducible modulation of flow conditions that rapidly leads to atherosclerosis has been one of the main factors limiting *in vivo* studies. Pioneering work by Krams *et. al*. and others showed that *d-flow* can be induced in the carotids by using a perivascular collar with a tapering lumen that induces a high shear stress field followed by regions of low shear stress and oscillatory shear stress upstream and downstream of the device, respectively, that ultimately results in atherosclerosis suggesting that *d-flow* can induce atherosclerosis^18–20^.

Recently, we showed that partial ligation of the mouse carotid artery causes *d-flow* and atherosclerosis development within two weeks, thus directly demonstrating the causal relationship between *d-flow* and atherosclerosis^21^. We also developed a novel method of obtaining carotid endothelial-enriched RNAs isolated from both the flow-disturbed left (LCA) and the contralateral, undisturbed right common carotid artery (RCA)^22^. Using this mouse model and endothelial RNA preparation technique, we reported a microarray study that identified novel flow-sensitive (also known as mechanosensitive) genes (mRNAs) in the mouse carotid endothelium. We used a whole-genome microarray study using mouse endothelial RNAs isolated from the flow-disturbed LCA and the undisturbed RCA. We found 62 and 523 genes that changed significantly by 12 and 48 h after ligation, respectively. The results were validated by quantitative PCR (qPCR) for 44 of 46 tested genes at the 48 h timepoint. This array study not only validated the well-known flow-sensitive genes, such as *Klf2*, *eNOS*, and *BMP4,* but also identified novel ones, including *lmo4*, *klk10*, and *dhh.*

Gene expression and regulation is a complex process and is orchestrated by transcriptional, post-transcriptional, and epigenetic regulators^1^. miRNAs have recently emerged as key regulators of gene expression and have been implicated in normal, as well as pathophysiological, events. To have a holistic idea of the gene expression landscape of the endothelium exposed to different flow conditions, more recently, we used the same experimental model and endothelial-enriched RNA collection method and identified a set of novel flow-sensitive miRNAs which are induced by *d-flow* in endothelial cells^11^. Our *in vivo* microarray data showed that 45 (27 up- and 18 downregulated) miRNAs were altered by more than 50% in the LCA endothelium as compared to the RCA at 48 h post-ligation^11^. Using additional independent RNA samples, the miRNA array data was validated by qPCR for the top 10 most flow-sensitive miRNAs (5 up-, 5 down-regulated miRNAs at 48 h post-ligation): upregulated (miR-330*, 712, 699, 223 and 770-5p) and down-regulated (miR-195, 30c, 29b, 26b and let-7d) miRNAs^11^. To determine whether these flow-sensitive miRNAs that were identified *in vivo* responded specifically to changes in shear stress, we tested expression of these miRNAs *in vitro* using immortalized mouse aortic endothelial cells (iMAECs) that were subjected to LS or OS, mimicking *s-flow* and *d-flow* conditions *in vivo*, respectively. Interestingly, most of the miRNAs that were upregulated in the endothelium by *d-flow* both *in vivo* were also found to be upregulated in iMAECs subjected to 24 h of OS *in vitro*. Collectively, using this experimental model and RNA collection method, we generated the ‘transcriptomic’ and ‘miRNomic’ profile of endothelial cells exposed to *d-flow* and *s-flow*, respectively^2,11^.

miRNAs are intricately involved in gene expression regulation and act by guiding the RNAi-induced silencing complex (RISC) to partially complementary sequences in target mRNAs to suppress gene expression either by translational inhibition or mRNA degradation^23–26^. miRNAs recognize target sites on the mRNA by base pairing at their 5' end called the seed region^27^ and that other factors, such as additional base pairing at the 3' end^28^, target site accessibility^29^, target site location and AU content around the target site, further contribute to target gene recognition^30^. These factors, as well as the evolutionarily conserved target sites (for conserved miRNAs), have been used to predict target sites of miRNAs^31^. Nevertheless, current prediction methods are important initial steps in miRNA target identification, however this approach is hampered by a significant number of false positives and false negatives^32^ and requires experimental validation. Therefore, deciphering and validating the accurate targets of miRNA continues to be one of the most difficult steps in miRNA research, but is crucial to understand its function and for the diagnostic and therapeutic applications.

To overcome these difficulties, we used two genome-wide datasets (mRNA microarray and miRNA microarray) to compare the expression values of miRNAs and mRNAs in endothelial cells *in vivo*. This approach helped us to narrow down the number of predicted gene targets of miRNAs significantly. For example, one of the most flow-sensitive miRNAs—miR-712 (upregulated by *d-flow*), was predicted to target more than ~9,000 genes by using the miR-walk prediction program. However, by cross-referencing these ~9,000 predicted target genes to our gene array data, we were able to narrow down the potential gene targets to 37 genes^11^. These small number of genes were then experimentally validated leading to discovery of tissue inhibitor of metalloproteinase 3 (TIMP3)^11^. Using this approach, we were able to systematically analyze the role of flow-sensitive miRNA (miR-712) in *d-flow* induced atherosclerosis. Here, we describe these two datasets (mRNA microarray and miRNA microarray studies) in depth that were generated from our two independent studies^2,11^. These include the data from our flow-sensitive mRNA array and miRNA array at two different time points (dataset 1 and dataset 2). The detailed description of these datasets should be helpful for other investigators. Determining the functional importance of these novel flow-sensitive genes and miRNAs may provide important insights into understanding vascular biology and atherosclerosis.

## Methods

These methods are expanded from previous descriptions in Son *et al*.^11^ and Ni *et al.*^2^

### Animals

6–8-week-old male C57Bl6 mice were used to collect endothelial-enriched total RNAs throughout the study. All animal experiments and surgical procedures were performed according to protocols approved by Institutional Animal Care and Use Committee (IACUC) at Emory University.

### Study design

We previously showed that partial ligation of the mouse carotid artery causes *d-flow,* which is sensed by the endothelial cells, leading to robust and rapid atherosclerosis development^21^. In order to understand the mechanisms by which flow regulates atherosclerosis, we performed microarray studies to determine the mRNA and miRNA expression profiles in response to different flow patterns: *d-flow* versus *s-flow*. To ensure that we identify both the early and late response genes that change due to *d-flow*, we chose two time points for RNA collection: 12 and 48 h post-ligation. We did not use time points earlier than 12 h as data at earlier time points could be an artifact due to the effect of anesthesia, surgical procedure, and the time required for change in gene expression. We also did not use time points later than 48 h, as there is increased immune cell infiltration after 48 h time point, especially in ApoE^−/−^ mice^2,33^.

### Partial carotid ligation surgery

**Figure 1:**
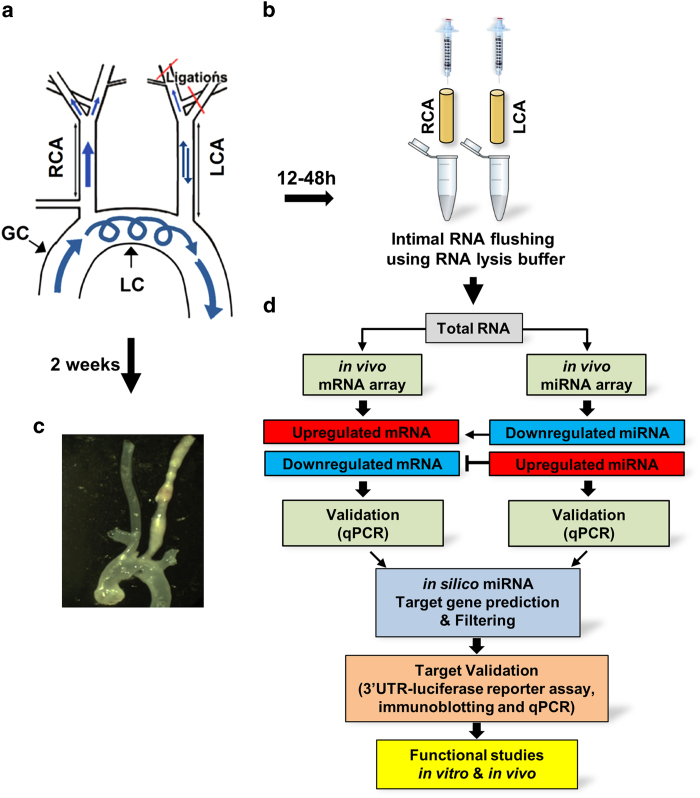
(**a**) The diagram shows naturally occurring *d-flow* (lesser curvature, LC) and *s-flow* regions (greater curvature, GC) in the aortic arch. Also shown is the surgically induced *d-flow* in the partial carotid ligation model in which three of the four caudal branches of the left common carotid artery (LCA) are ligated, while the contralateral right common carotid artery (RCA) remains untouched in order to serve as an internal control. (**b**) The scheme shows the intimal RNA extraction step that enables collection of endothelial-enriched RNAs from the mouse carotids. Using an insulin syringe with a 29G needle and 150 μl QIAzol lysis buffer (Qiagen) and carefully flushing the intima with QIAzol lysis buffer into an Eppendorf tube. Intima eluate or the leftover (media+adventitia) are then used for total RNA isolation. (**c**) Representative image of the ApoE^−/−^ mouse carotids showing plaque in the LCA after 2 weeks of high-fat diet confirming disease status, while the RCA remains plaque-free. (**d**) The scheme shows our approach to discover potential, flow-sensitive miRNAs-target gene interactions. Using a simplified hypothesis that the upregulated miRNA will negatively regulate its target gene and vice versa, we initially generated a predicted target gene list for highly differentially regulated miRNAs and then compared this putative target gene list to the list of flow-sensitive mRNAs (that followed an inverse relation with the miRNAs) in the mRNA array. Using this approach, we identified two potential targets for miR-712 as TIMP3 and RECK.

Survival surgeries were performed on anesthetized mice (6- to 8-week-old male C57Bl/6 mice) with partial ligation on left carotid arteries (LCA)^21^. Briefly, three of four caudal branches of LCA (left external carotid, internal carotid, and occipital artery) were ligated using 6-0 silk suture, whereas the superior thyroid artery was left intact (Figure 1a). The contralateral RCA was left undisturbed and served as internal control. Following surgery, analgesic buprenorphine (0.1 mg kg^−1^) was administrated. Mice were monitored post-surgery and were allowed to recover. Post procedure, *d-flow* in the LCA of all animals was confirmed by ultrasonography to verify the success of partial ligation surgery^21^. To study atherosclerosis, ApoE^−/−^ mice were fed a high-fat diet for 2 weeks after surgery (Figure 1c).

### RNA collection

Endothelial-enriched RNA was collected from the carotids by flushing the lumen quickly with Qiazol^22^. Briefly, LCA and RCA were quickly flushed with 150 μl of QIAzol lysis reagent (Qiagen) using 29 G insulin syringe into a microfuge tube (Figure 1b). The eluate was then used for total intimal RNA isolation using the miRNeasy mini kit (Qiagen) following manufacturer’s protocol. DNase I was used to remove DNA contamination in the RNA samples.

### Initial quality control

After the RNA extraction, each sample was tested using Agilent BioAnalyze NanoChip to check the quantity and quality of total RNA. To test for endothelial enrichment of the intimal RNA extracted, we performed qPCR for endothelial (PECAM-1) and non-endothelial markers, α-SMA (smooth muscle cell marker) and CD11b (leukocyte marker). We also checked the expression levels of well-known flow-sensitive genes, such as *eNOS, klf2*, and *BMP4*. All samples that passed our initial quality control check, both biologically and technically, were used for subsequent microarray study.

### mRNA microarray and data analysis

Endothelial-enriched total RNAs were obtained from LCA and RCA at 12 and 48 h after ligation. Endothelial-enriched RNAs from 3 LCAs or RCAs were pooled to obtain approximately 30 ng of total RNA. Each sample was linearly amplified by WT-Ovation RNA amplification system (NuGEN) and used for the microarray study using MouseWG-6 v2 Expression BeadChip array with 45, 281 probes (Illumina) at the Emory Biomarker Service Center. After hybridization, BeadChips were scanned on the Illumina BeadArray Reader to determine the probe fluorescence intensity. The raw probe intensities were then normalized by the quintile normalization algorithm using the GenomeStudio software from Illumina. The normalized microarray data were statistically analyzed by Significance Analysis of Microarrays software (SAM 3.0)^34,35^. The genes that were identified as having differential expression between the LCA and RCA were those that showed greater than 1.5-fold change at a false discovery rate (FDR) of less than 10%. The lists of differentially expressed genes were tested for statistical significance.

### miRNA microarray and data analysis

RNA samples from three mice were pooled in order to increase the amount of total RNA to 30 ng and reduce the variability from individual animals. Paired samples of LCA and RCA from the same sets were labeled as the identical series number in order for compare the expression of mRNA and miRNA expression from the RCA and LCA since the RCA served as an internal as well as the baseline control. Illumina’s GenomeStudio, data analysis software platform was used for analyzing data obtained from microarrays. The GenomeStudio Gene Expression Module, included with the Illumina MicroRNA Expression Profiling Assay, was used for analyzing miRNA expression data from scanned microarray images collected from the BeadArray Reader. Experimental performance was assessed based on built-in controls that accompany each experiment. The resulting GenomeStudio expression results were exported and further analyzed.

### Computational *in silico* analyses

We compared a list of putative miRNA targets generated by multiple miRNA target prediction algorithms (miRWalk) and compared this to the list of downregulated flow-sensitive genes^2^. Thus, we narrowed down the predicted gene targets of miRNA to a workable list of genes that were downregulated in the mRNA microarray. This comparison of upregulated miRNAs and concomitantly downregulated genes from the same experimental system allowed us to identify the possible *d-flow* dependent, miRNA-gene interaction networks in an *in vivo* setting. Also, this filtering approach significantly reduced the workable target list for further experimental evaluation (Figure 1d). The potential target genes were functionally annotated using DAVID (http://david.abcc.ncifcrf.gov/) and the Kyoto Encyclopedia of Genes and Genomes (KEGG) pathways^36^ to generate a *d-flow* induced miRNA interactome. We were focused on identifying key network genes which had a cascading effect downstream (e.g. TIMP3). Additional interactions among TIMP3-associated genes were identified through physical protein–protein interaction (ftp://ftp.ncbi.nih.gov/gene/GeneRIF/)^37,38^, transcription factor-target relations^39^, and enriched by adding non-flow-sensitive genes with known associations to TIMP3 using Information Hyperlinked over Proteins (iHOP)^40^. This analytical approach resulted in identification of important miRNA-target gene and affected signaling pathway downstream.

## Data Records

There are two data records deposited in the Gene Expression Omnibus database, GSE20741 (Data Citation 1) and GSE52243 (Data Citation 2) for mRNA and miRNA expression, respectively^2,11^. For the GSE20741, we used the RNAs from 3 RCA and 3 LCAs, respectively, for two time points, 12 and 48 h (GSM520561 to GSM520566). Genome-wide analysis of flow-sensitive genes using mouse carotid artery endothelium exposed to disturbed flow. The structured data are contained in tables indicating the gene transcript tested and its relative expression in the biological replicates of mouse artery endothelium. For the GSE52243 (miRNA expression), we used the RNAs from 6 RCA and 6 LCAs, respectively, for two timepoints, 12 and 48 h, each (GSM1261717 to GSM1261740).

## Technical Validation

**Figure 2:**
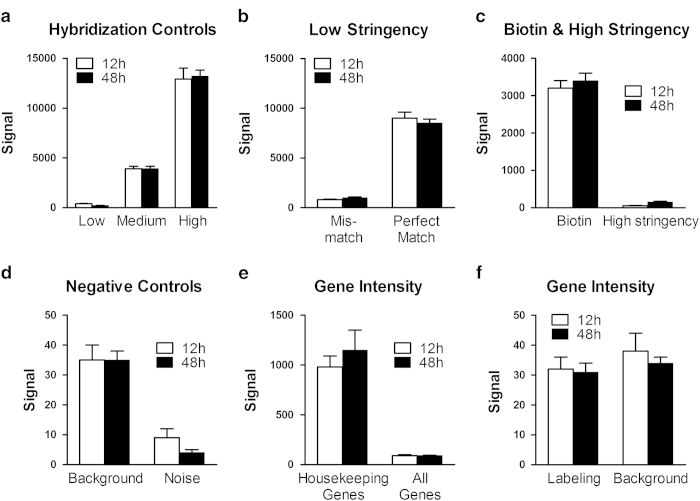
Control Summary plots for 12 and 48 h mRNA microarray. Illumina gene expressions BeadChips have internal control features to monitor data quality. The results of these controls as visualized by GenomeStudio by selecting the control probe profile are shown. (**a**) Hybridization controls. (**b**) Low stringency probes (**c**) Biotin and high stringency controls (**d**) Negative controls (**e**) Gene intensity controls for housekeeping genes and (**f**) gene intensity controls for labeling and background. Data represents fluorescence signal intensities as means±s.d.

**Figure 3:**
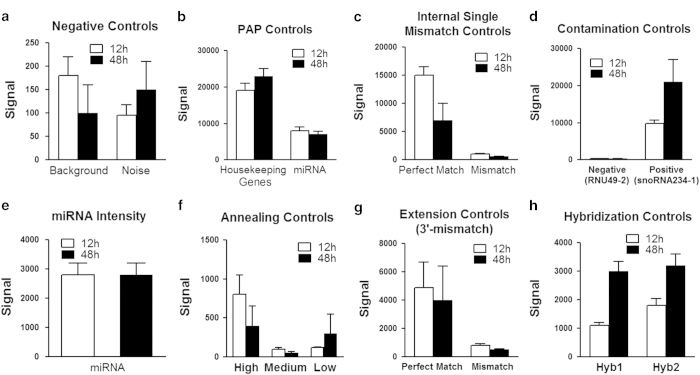
Control Summary plots for 12 and 48 h microRNA microarray. Illumina gene expression BeadChips have internal control feature to monitor data quality. (**a**) Negative controls. (**b**) Polyadenylation (PAP) Controls (**c**) Internal Single mismatch controls. (**d**) Contamination Detection Controls. (**e**) miRNA intensity controls. (**f**) Annealing controls. (**g**) Extension controls. (**h**) Array Hybridization Controls. Data represents fluorescence signal intensities as means±s.d.

**Figure 4:**
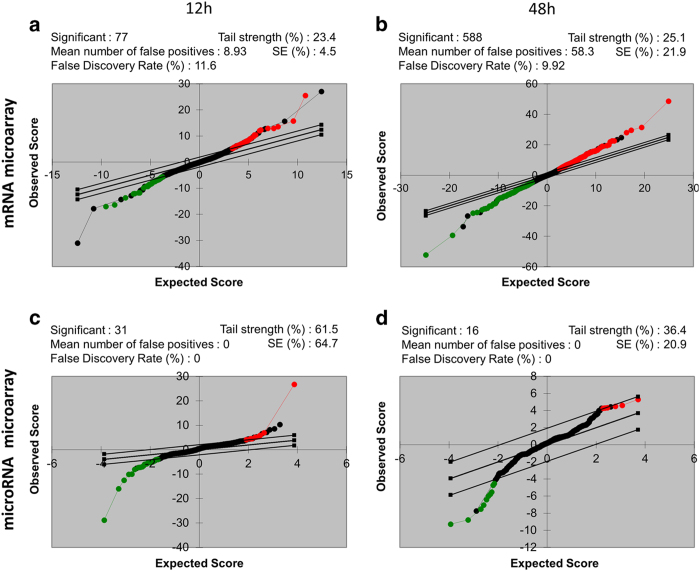
Pair wise analysis by a modified t-test of SAM and the validation of the cDNA microarray data. Scatter plots of the observed score versus the expected score for the mRNA microarrays at (**a**) 12 h and (**b**) 48 h timepoints and for the miRNA microarrays at (**c**) 12 h and (**d**) 48 h timepoints are plotted. Significance analysis of microarrays (SAM) identifies significantly differentially expressed genes. The solid parallel lines indicate the region where the observed relative difference is identical to the expected relative difference. The potentially significant genes and miRNAs are indicated by red circles (up-regulated genes or miRNAs) and green circles (down-regulated genes or miRNAs), respectively.

To obtain a comprehensive assessment of data quality, we used a broad panel of statistics and metric analysis. To have confidence in our microarray data, we used biological replicates instead of mere technical replicates. Post processing, GenomeStudio GX module was used to generate a report, record the control values after every run, and analyze control measurement data (which provides a basis for assessing the validity of the results) of the mRNA and the microRNA. Likewise, the post microarray analysis using Significance Analysis of Microarrays software (SAM 3.0)^34,35^ showed the scatter plots of the observed score versus the expected score for the mRNA and miRNA microarrays at 12 and 48 h time points. All the metrics were used to determine that the assay performance was satisfactory (Figs 2, 3 and 4).

### Microarray study controls

Genome Studio control features are either sample-independent or sample-dependent. The sample-independent metrics make use of the oligonucleotide spiked into the hybridization solution. Poor performance measures by these controls could indicate a general problem with hybridization washing or staining. The sample-dependent metrics are based on measurements from the actual sample of interest. Poor performance of these controls may indicate problems related to the sample or labeling. Normal variations in control plot values arise due to incidental factors such as system setup, sample origin, and BeadChip type. These factors make it difficult to determine data quality by comparison to a specific value for each QC metric. To minimize these factors, relative, rather than absolute control values, should be used to check the quality of the assay.

For the mRNA microarray, the following internal control probes were used. These include the (1) hybridization controls (Figure 2a). The array hybridization controls test the hybridization of single-stranded assay products to the array beads. The controls consist of 50-mer oligos labeled with Cy3 dye included in the hybridization reagent. The probes in this control category correspond to Cy3-labeled oligonucleotides. Following successful hybridization, they produce a signal independent of both the cellular RNA quality and success of the sample prep reactions. Target oligonucleotides for the Cy3 Hybridization controls are present at concentrations of low, medium, or high, yielding gradient hybridization responses. (2) Low stringency probes correspond to the medium- and high concentration Cy3 Hybridization control targets (Figure 2b). In this case, each probe has two mismatch bases distributed in its sequence. If stringency is adequate, these controls yield very low signal. If stringency is too low, they yield signal approaching that of their perfect match counterparts in the Cy3 Hybridization control category. (3) Biotin and high stringency controls (Figure 2c). Also known as signal generation controls. This category consists of two probes with complementary biotin-tagged oligonucleotides present in the hybridization buffer. Successful secondary staining is indicated by a positive hybridization signal from these probes. (4) Negative controls. This category consists of probes of random sequence selected to have no corresponding targets in the genomes (Figure 2d). The mean signal of these probes defines the system background. This is a comprehensive measurement of background, representing the imaging system background (5) Gene intensity controls for housekeeping genes. The intactness of the biological specimen can be monitored by the housekeeping gene controls and gene intensity for labeling and background (Figure 2e,f).

For the microRNA microarray, the following internal control probes were used. These include the (1) Negative controls. This category consists of query oligos targeting random sequences that do not appear in the mouse genome. The mean signal of these probes defines the system background (Figure 3a). (2) Polyadenylation (PAP) Controls (Figure 3b). Polyadenylation control oligos detect transcripts of a set of highly expressed housekeeping genes that already contain a stretch of poly-A sequence. If the polyadenylation process is ineffective, they will not be amplified and will not show signal. (3) Internal Single mismatch controls (Figure 3c). The mismatch controls measure the specificity of extension in the second strand cDNA synthesis step by comparing the signal intensity of perfectly matched oligos versus oligos with an internal mismatch. The perfectly matched query oligo should show signal, while the mismatched one should show dramatically decreased signal. (4) Contamination Detection Controls (Figure 3d). The PCR contamination detection controls described in this section are designed against mouse small nuclear RNA (SnoRNAs) and are divided into two types. Only one type is added to each oligo pool for the MicroRNA Assay. When a single MAP is run, only one contamination control type should have high signal. If both contamination control types have high signal, indicating significant contamination may have occurred. (5) miRNA intensity controls (Figure 3e). The BeadArray Reader measures fluorescence intensity at each addressed bead location. The intensity of the signal corresponds to the quantity of the respective miRNA in the original sample. (6) Annealing Controls (Figure 3f). Annealing controls test the efficiency of annealing microRNA Specific Oligos (MSOs) with different T_ms_ to the same cDNA target. The higher T_m_ MSO should always have higher signal than the lower T_m_ MSO. (7) Extension controls (Figure 3g). The extension controls measure the specificity of extension in the second strand cDNA synthesis step by comparing the signal intensity of perfectly matched oligos versus oligos with a mismatch at the 3′-end. The perfectly matched query oligo should show signal, while the mismatched one should show dramatically decreased signal. (8) Array Hybridization Controls (Figure 3h). Hybridization controls test the hybridization of single-stranded assay products in the array.

### Validation of experimental animal model

The methodology presented in this dataset has been validated and corroborates with previous publications from our lab and others^2,21,22,33,41–46^. The RCA serves as the internal control. Since both the carotid arteries are straight (and thus are exposed to laminar flow), these common carotids do not naturally develop atherosclerosis; however, only the partially ligated LCA experiences a blood flow change to reverse and oscillatory flow, and subsequently develops atherosclerotic plaques (Figure 1d). We compared the well-known flow-sensitive genes from the literature and all these genes were validated in the array performed using our *in vivo* experimental model. Therefore, our experimental system is a reliable method for identifying flow-sensitive genes.

### Biological replicates

A total of 108 mice were used for both microarray studies. For the mRNA microarray, we used 3 biological replicates for each RCA and LCA sample at a given time point. Further, each sample itself came from a pool of 3 mice carotid artery endothelial RNAs. For the miRNA microarray, we used 6 biological replicates for each RCA and LCA sample at each time point and each sample was composed of endothelial RNAs from a pool of 3 mouse carotid arteries.

### Validation of results

**Figure 5:**
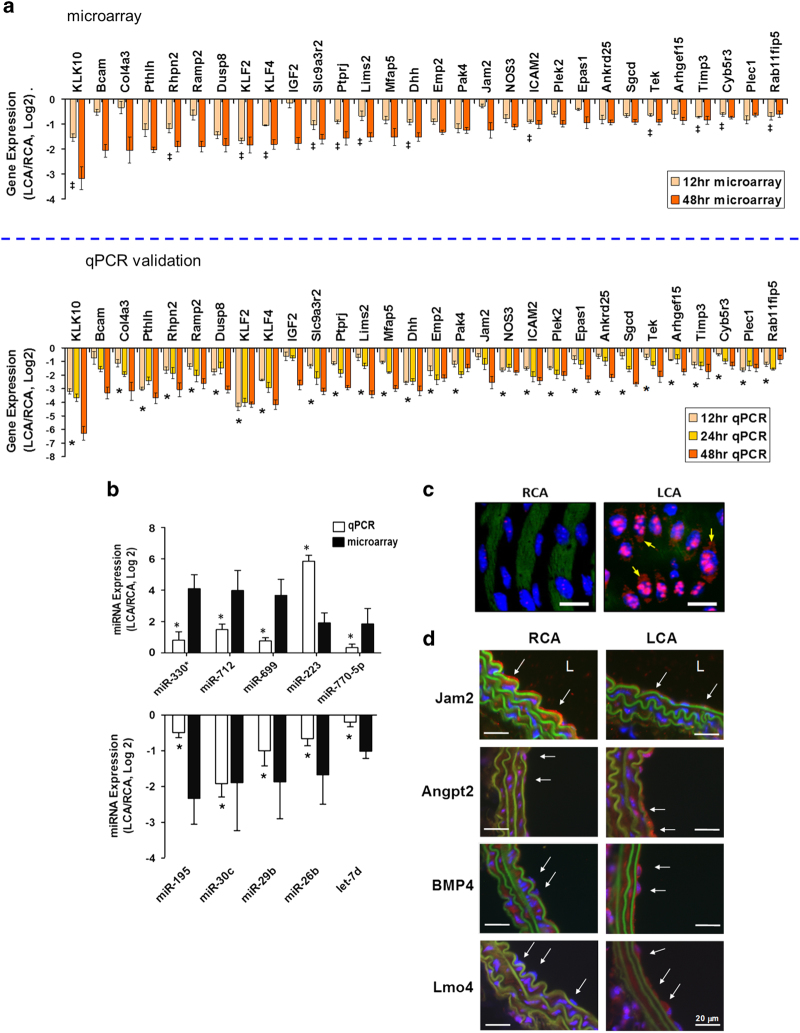
(**a**) Validation of mechanosensitive genes by qPCR. Total RNAs from intima of LCA or RCA at 12, 24, and 48 h post-ligation were collected and 3 different mouse carotids were pooled, representing a total of 9 to 15 mice. Genes selected for qPCR analyses were based on microarray results (top panel). Bottom panel shows change in gene expression for the corresponding genes validated from independent samples as fold change in LCA over RCA in log2 scale (mean *s.e.m.; *n* =3–5). *Gene expression change was also statistically significant in LCA versus RCA (*P*<0.05) at 12 and 48 h timepoint. All genes shown were significant at FDR<10% at 48 h. ‡Gene expression change also statistically significant in LCA versus RCA at 12 h. Data are mean s.e.m. (*n*=3). (**b**) Validation of miRNA microarray by qPCR. Using the same experimental plan as above, the qPCR study validated the microarray results for 5 up-regulated (miR-712, -330*, -699, -223, and 770-5p) and 5 down-regulated (miR-195, -30c, -29b, -26b and let-7d) miRNAs at the 48 h time point (*n*=5 each, data shown as mean±s.e.m; *P<0.05 (paired *t-*test). (**c**) Validation of prototypical miRNA miR-712 by *in situ hybridization* LCA and RCA obtained at 2-days post ligation from C57Bl6 mice were subjected to fluorescence *in situ* hybridization using digoxigenin-labeled miR-712 probe and anti-digoxigenin antibody, which was detected by tyramide signal amplification method using Cy-3 and confocal microscopy (shown in red), (*n*=6). Blue: DAPI nuclear stain; Green: auto-fluorescent elastic lamina; Arrows indicate cytosolic miR-712. White scale bars=20 μm. (**d**) Validation of key flow sensitive genes (Jam2, Angpt2, BMP4, and Lmo4) by immunofluorescence. LCA and RCA were collected 2 days after ligation from C57Bl6 mice. Paraffin sections were stained with specific antibodies for Jam2 (A), Angpt2 (B), BMP4 (C), and Lmo4 (D). Nuclei counterstained with Hoechst (blue). Disturbed flow in LCA decreases protein expression of Jam2 while upregulating Angpt2, BMP4, and Lmo4. Arrows indicate the protein expression in endothelial cells. L indicates lumen. Images are representative of *n*=4 mice. This figure was originally published in Blood. Ni *et al.*^2^ © the American Society of Hematology, and in Nature Communications. Son* et al.*^11^

The results from the microarray studies were validated by selectively testing the expression of highly upregulated and downregulated mRNAs and microRNAs using additional samples from mouse carotid artery endothelium (Figure 5a,b). Furthermore, we used immunohistochemistry and *in situ* hybridization to further validate the expression of selected genes and microRNAs using additional samples (Figure 5c,d). These findings further confirmed the array results and justified the reliability of our microarray data.

### Confirming disease status

Induction of *d-flow* in the LCA due to partial ligation surgery induces atherosclerosis within 2 weeks when the animals are fed a high-fat diet. To confirm the disease process, we allowed some of the ApoE^−/−^ mice to remain on a high-fat diet for 2 weeks after partial ligation surgery. All the animals developed atherosclerotic lesions in their LCA while the RCA remained plaque-free (Figure 1c).

## Usage Notes

There are several potential uses for this dataset. Most data showing flow-dependent endothelial gene changes have been generated using endothelial cells cultured *in vitro* and using various shear stress devices. Endothelial cells are well-known to undergo genotypic and phenotypic drift during *in vitro* culture. It was previously estimated that up to 45% of all mechanosensitive genes are either lost or significantly dysregulated during static culture without flow, compared to that of *in vivo* conditions^2^. Given the limited number of *in vivo* studies carried out *in vivo* using mouse and pigs^11,15^, these mechanosensitive endothelial genes that are directly obtained from the study like ours are extremely valuable. These *in vivo* flow sensitive genes (mRNAs and miRNAs) which are differentially regulated by *d-flow* and *s-flow* can be compared to *in vitro* data. Further mining of these two key data sets (*d-flow* induced miRNAs and *d-flow* altered genes) could provide insight into the underlying mechanisms of *d-flow* induced atherosclerosis. Additionally, the datasets could be analyzed to identify key hub genes and important gene networks that are important in the pathophysiological process of atherosclerosis.

Furthermore, public domain research tools such as DAVID and commercially available tools such as Ingenuity IPA and Meta-Core, can be used to analyze these datasets to generate new metadata and subsequent hypothesis-driven research as we have previously shown^11^.

## Additional information

**How to cite this article:** Kumar, S. *et al.* Flow-dependent regulation of genome-wide mRNA and microRNA expression in endothelial cells *in vivo*. *Sci. Data* 1:140039 doi: 10.1038/sdata.2014.39 (2014).

## Supplementary Material
